# Antibiotic resistance trends for common bacterial aetiologies of childhood diarrhoea in low- and middle-income countries: A systematic review

**DOI:** 10.7189/jogh.13.04060

**Published:** 2023-07-21

**Authors:** Raghavee Neupane, Myra Bhathena, Gopika Das, Elizabeth Long, Jennifer Beard, Hiwote Solomon, Jon L Simon, Yasir B Nisar, William B MacLeod, Davidson H Hamer

**Affiliations:** 1 Department of Global Health, Boston University School of Public Health, Boston, Massachusetts, USA; 2 Department of Maternal, Newborn, Child and Adolescent Health and Ageing, World Health Organization, Geneva, Switzerland; 3 Section of Infectious Diseases, Department of Medicine, Boston University Chobanian & Avedisian School of Medicine, Boston, Massachusetts, USA; 4 Center for Emerging Infectious Diseases Policy and Research, Boston University, Boston, Massachusetts, USA; 5 National Emerging Infectious Disease Laboratory, Boston University, Boston, Massachusetts, USA; 6 Friedman School of Nutrition Science and Policy, Tufts University, Boston, Massachusetts, USA

## Abstract

**Background:**

Diarrhoea is the second most common cause of death among children under the age of five worldwide. The World Health Organization (WHO) recommends treating diarrhoea with oral rehydration therapy, intravenous fluids for severe dehydration, and zinc supplements. Antibiotics are only recommended to treat acute, invasive diarrhoea. Rising antibiotic resistance has led to a decrease in the effectiveness of treatments for diarrhoea.

**Methods:**

A systematic literature review in PubMed, Web of Science, and EMBASE was conducted to identify articles relevant to antibiotic-resistant childhood diarrhoea. Articles in English published between 1990 to 2020 that described antibiotic resistance patterns of common pathogens causing childhood diarrhoea in low- and middle-income countries were included. The studies were limited to papers that categorized children as 0-5 years or 0-10 years old. The proportion of isolates with resistance to major classes of antibiotics stratified by major WHO global regions and time was determined.

**Results:**

Quantitative data were extracted from 44 articles that met screening criteria; most focused on children under five years. *Escherichia coli* isolates had relatively high resistance rates to ampicillin and tetracycline in the African (AFR), American (AMR), and Eastern Mediterranean Regions (EMR). There was moderate to high resistance to ampicillin and third generation cephalosporins among *Salmonella* spp in the AFR, EMR, and the Western Pacific Region (WPR). Resistance rates for ampicillin, co-trimoxazole, and chloramphenicol for *Shigella* in the AFR started at an alarmingly high rate ( ~ 90%) in 2006 and fluctuated over time. There were limited antibiotic resistance data for *Aeromonas*, *Yersinia*, and *V. cholerae*. The 161 isolates of *Campylobacter* analysed showed initially low rates of fluoroquinolone resistance with high rates of resistance in recent years, especially in the Southeast Asian Region.

**Conclusions:**

Resistance to inexpensive antibiotics for treatment of invasive diarrhoea in children under ten years is widespread (although data on 6- to 10-year-old children are limited), and resistance rates to fluoroquinolones and later-generation cephalosporins are increasing. A strong regional surveillance system is needed to carefully monitor trends in antibiotic resistance, future studies should include school-aged children, and interventions are needed to reduce inappropriate use of antibiotics for the treatment of community-acquired, non-invasive diarrhoea.

**Registration:**

This systematic review was registered in Prospero (registration number CRD42020204004) in August 2020.

As one of the leading causes of child mortality, diarrhoeal diseases pose a significant global burden, disproportionately affecting children in low- and middle-income countries (LMICs) [1]. In 2017, diarrhoeal diseases were responsible for approximately 8% of worldwide deaths among post-neonatal children aged 1 to 59 months, with 525 000 children dying yearly [1]. Managing childhood diarrhoea involves rehydration therapy with oral rehydration solution (ORS), intravenous fluids for severe dehydration, and zinc supplements [2].

Antibiotic resistance, common in LMICs, contributes to the failure of well-established treatment options for childhood diarrhoea, leading to a cascade of events compounding the illness [3]. In addition to complicating the management of invasive (bloody) diarrhoea, rising antibiotic resistance is associated with an increased risk of childhood morbidity and mortality [4] and increased health care costs.

The World Health Organization (WHO) does not recommend antibiotics for the treatment of non-bloody diarrhoea (also referred to as non-invasive diarrhoea) [5], with the notable exception of non-invasive diarrhoea caused by *Vibrio cholerae* in patients hospitalized with severe dehydration or in those with underlying conditions that increase risk of severe disease [6]. The widespread inappropriate use of antibiotics for non-invasive diarrhoea has contributed to high levels of antibiotic resistance [7]. The WHO recommends ciprofloxacin as the first-line drug to treat invasive diarrhoea in addition to ORS [7].

Fluoroquinolone resistance has become increasingly recognized among common bacterial diarrhoeal pathogens such as *Campylobacter jejuni*, *Shigella* spp, non-typhoidal *Salmonella*, and toxin-producing strains of *Escherichia coli* in studies of travellers to South and Southeast Asia [8,9]. This suggests the potential reduced utility of ciprofloxacin for invasive diarrhoea in this region.

Past studies have primarily focused on children under five years old. While this analysis also evaluates changes in antibiotic resistance patterns between 1990-2020 among under-five children with acute diarrhoea, this systematic review primarily aims to identify the patterns and regional distribution of antibiotic resistance of select bacterial diarrhoeal pathogens in children ages 0-10 years to identify studies of the prevalence of antibiotic resistance among diarrhoeal pathogens in children ages 6-10 years.

## METHODS

We conducted a systematic literature review in PubMed, Web of Science, and EMBASE to identify relevant articles in the peer-reviewed literature. Our search strategy used the following MeSH terms: “drug resistance, microbial,” “*Shigella,*” “*Yersinia,*” “*Escherichia coli,*” “*Salmonella,*” “*Campylobacter,*” “*Plesiomonas,*” “*Aeromonas,*” “*Vibrio cholera,*” “diarrhoea,” “child,” and “infant.”

Inclusion and exclusion criteria were established before the search. We included observational studies, cohort studies, randomized controlled trials, experimental studies, and quasi-experimental studies published in English between 1990 to 2020 that described antibiotic resistance patterns of select pathogens causing childhood diarrhoea in LMICs. Studies were limited to papers that categorized children as 0-5 or 0-10 years old. Since most studies did not provide a breakdown by age, the results provided below are for all ages in the range of 0-10 years.

Multi-pathogen and single-pathogen studies which specifically measured the antimicrobial resistance patterns of *Shigella, E coli, Salmonella,* and *Campylobacter* species responsible for childhood diarrhoea were included. We also searched for antibiotic resistance studies that included the following organisms: *Yersinia, Aeromonas,* and *V. cholerae*.

All studies which involved children older than ten years or adults were excluded unless specific age group data could be accurately extracted. Case series and case reports were excluded. All studies which did not use the WHO definition for diarrhoea (defined as the passage of watery stools at least three times within one day [10] or which used inconsistent or inappropriate laboratory techniques were excluded.

Two of the authors (GD, EL) independently assessed the eligibility criteria of each article. A third researcher (RN) resolved discrepancies in article reconciliation. The reviewers independently screened titles and abstracts to determine their eligibility for inclusion. The full texts of the selected articles were retrieved and separately reviewed by both researchers. Finally, the 44 chosen articles were reconciled, and quantitative data were extracted. If only one study could be found, the proportion of resistant isolates was recorded, whereas if two or more studies were identified with data from the same time, the mean proportion of antibiotic resistance was recorded.

## RESULTS

**Figure 1:**
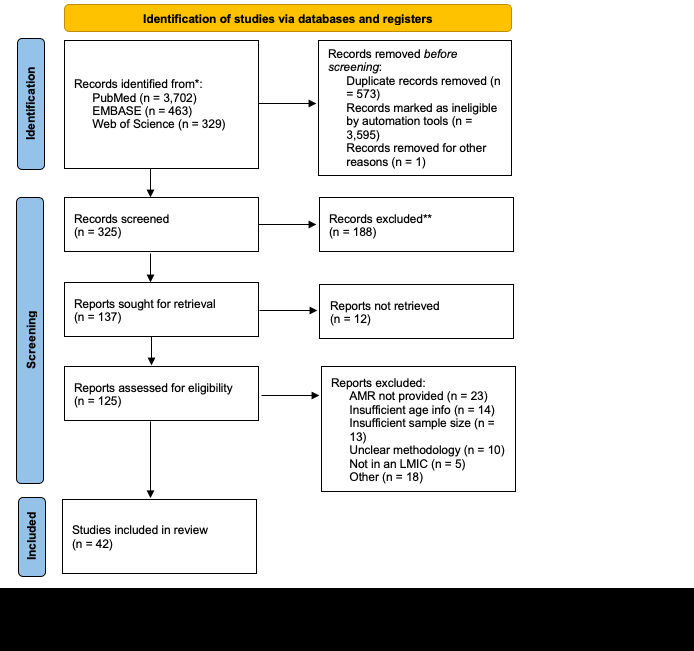
PRISMA flow diagram of article extraction for systematic review of antimicrobial resistance (AMR) in childhood diarrhoea in low- and middle-income countries (LMIC) [11].

There were 42 articles identified by the literature search (**Figure 1**). The largest number of studies included *E coli,* followed by *Salmonella* spp and *Shigella* spp (**Table 1**). There were relatively few studies of *Campylobacter* spp and minimal data for *Aeromonas, Yersinia*, and *V. cholerae*. In general, there were more studies from the African Region (AFR) and the Eastern Mediterranean Region (EMR) than from the South-East Asian Region (SEAR), American Region (AMR), and Western Pacific Region (WPR) of WHO.

**Table 1:** Summary of the number of studies and isolates categorized by World Health Organization regions for children aged 0-10 years

Pathogen	Number of isolates in AFR (number of studies)	Number of isolates in AMR (number of studies)	Number of isolates in EMR (number of studies)	Number of isolates in EUR (number of studies)	Number of isolates in SEAR (number of studies)	Number of isolates in WPR (number of studies)	Isolates (total number of studies)
E coli	3350 (7)	3361 (8)	3024 (13)	-	164 (2)	378 (2)	10 277 (32)
Shigella	188 (5)	56 (1)	122 (5)	289 (1)	344 (5)	62 (1)	1061 (18)
Salmonella	705 (8)	-	139 (4)	-	38 (1)	502 (5)	1384 (18)
Aeromonas	-	-	52 (1)	-	-	10 (1)	62 (2)
Yersinia	-	-	24 (2)	-	-	-	24 (2)
V. cholerae	50 (1)	-	-	-	-	-	50 (1)
Campylobacter	60 (3)	-	-	-	17 (1)	84 (3)	161 (7)

AFR – African Region, AMR – Region of the Americas, EMR – Eastern Mediterranean Region, EUR – European Region, SEAR – South-East Asian Region, WPR – Western Pacific Region

Full details of all articles included in the systematic review, including study site, year(s) of data collection, age range, and the number of isolates, are provided in Web Table. Data are presented as the proportion of isolates with resistance to major classes of antibiotics stratified by major WHO global regions and time.

### E coli

**Figure 2:**
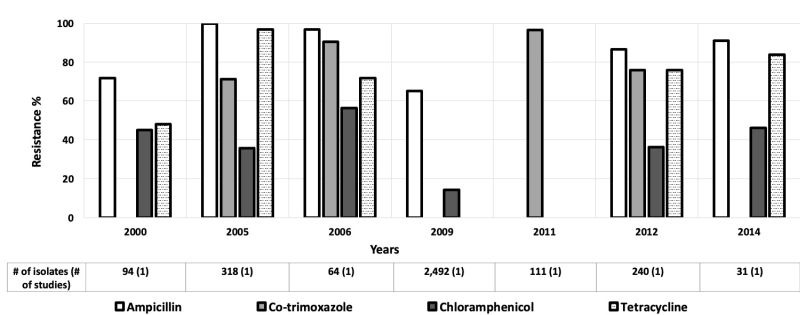
Antibiotic resistance among *Escherichia coli* isolates in the African Region (AFR) by time period. The number of isolates tested for time period and number of studies are shown below the relevant time period.

**Figure 3:**
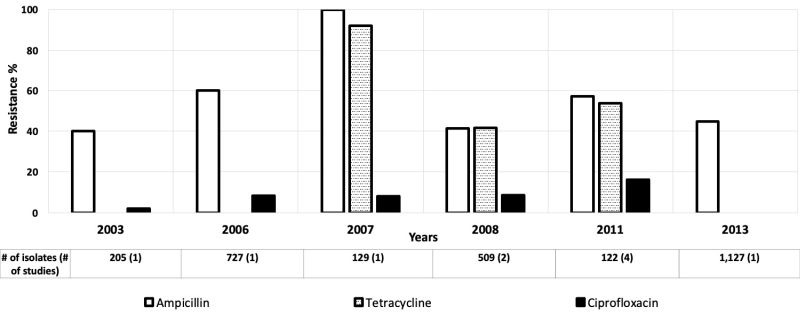
Antibiotic resistance among *Escherichia coli* isolates in the American Region (AMR) by time period. The number of isolates tested for time period and number of studies are shown below the relevant time period.

**Figure 4:**
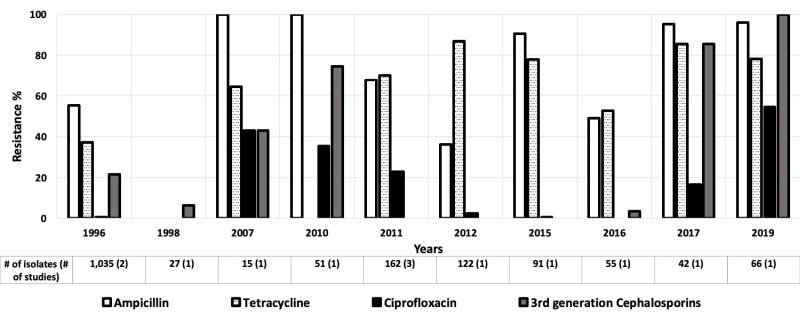
Antibiotic resistance among *Escherichia coli* isolates in the Eastern Mediterranean Region (EMR) by time period. The number of isolates tested for time period and number of studies are shown below the relevant time period.

Twenty-seven studies looked at antibiotic resistance among *E coli* isolates for children aged 0-5 years [12-38], and five provided data for children aged 0-10 years [39-43]. Resistance levels among *E coli* isolates varied widely, with relatively high rates of resistance to ampicillin and tetracycline in the AFR (**Figure 2**), AMR (**Figure 3**), and EMR (**Figure 4**). Only these regions were included because the data available for those regions were much more numerous than for the other regions.

Ciprofloxacin resistance increased from 2% to 9% in the AMR between 2003 and 2011 (**Figure 3**) [15,40]. Likewise, in the EMR, third generation cephalosporin resistance rose from low to moderate between 1996 and 2010, and then decreased drastically in 2016 (**Figure 4**) [15,20,44]. The third-generation cephalosporin resistance was 3.6% in 2016 in Hilla city, Iraq [20], whereas in Dhi Qar Governorate, Iraq, resistance to third-generation cephalosporin was 100% in 2019 [19] (**Figure 4**).

### Salmonella

**Figure 5:**
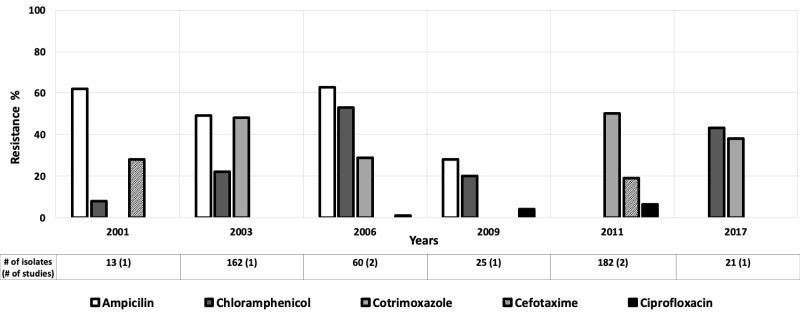
Antibiotic resistance among *Salmonella* isolates in the African Region (AFR) by time period. The number of isolates tested for time period and number of studies are shown below the relevant time period.

**Figure 6:**
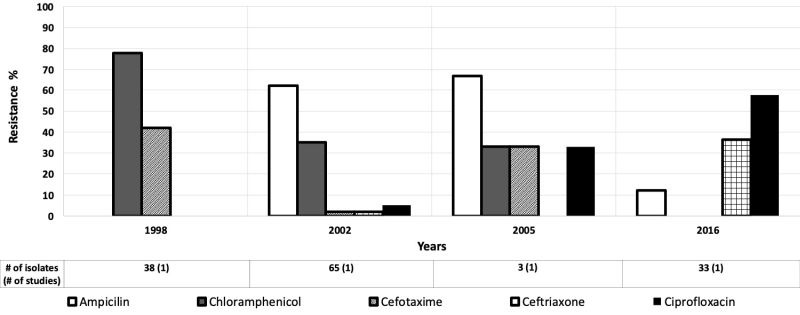
Antibiotic resistance among *Salmonella* isolates in the Eastern Mediterranean Region (EMR) by time period. The number of isolates tested for time period and number of studies are shown below the relevant time period.

**Figure 7:**
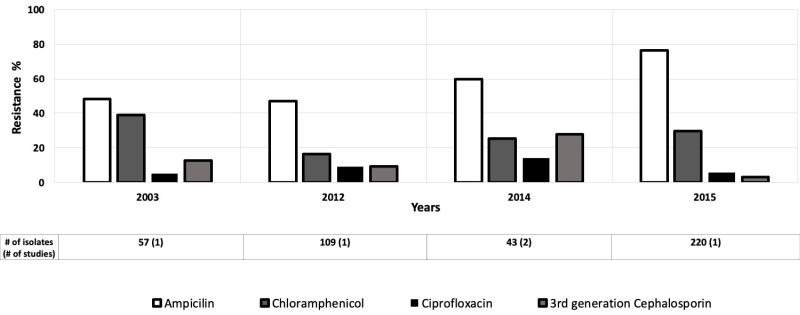
Antibiotic resistance among *Salmonella* isolates in the Western Pacific Region (WPR) by time period. The number of isolates tested for time period and number of studies are shown below the relevant time period.

Nine studies evaluated antibiotic resistance of *Salmonella* isolates in children aged 0-5 years [12,13,22,27,34,38-41] and four studies provided data for children aged 0-10 years [43,45-47]. Moderate to high resistance to ampicillin among *Salmonella* spp was found in the AFR, EMR, and WPR. Ampicillin resistance decreased from 62% to 28% from 2001 to 2009 in the AFR (**Figure 5**) [35,48], and from 62% to 12% in the EMR between 2002 and 2016 (**Figure 6**) [46,48]. But, between 2003 and 2015, ampicillin resistance increased from 48% to 77% in the WPR (**Figure 7**) [49,50]. Only these regions were included because of the data available for those regions were much greater than the other regions.

Ciprofloxacin resistance was generally low (<10%) among *Salmonella* isolates, with the notable exceptions of two studies in 2005 (33%) [51] and 2016 (58%) in the EMR [48]. The levels of resistance to third-generation cephalosporins were moderately elevated in WPR from 2003 to 2014 [49,52], except for a study in 2015 where the resistance to third generation cephalosporin decreased to 3% [50] (**Figure 7**).

### Shigella

**Figure 8:**
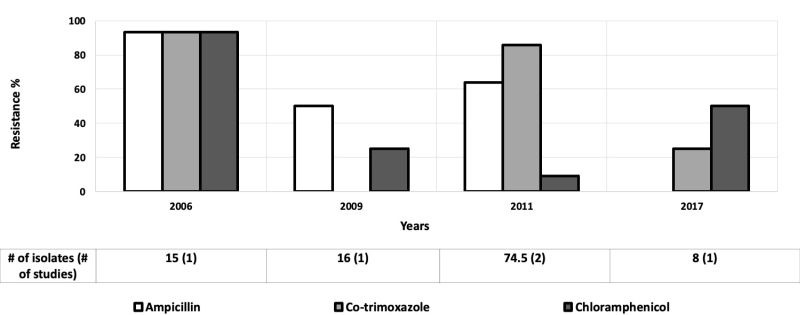
Antibiotic resistance among *Shigella* isolates in the African Region (AFR) by time period. The number of isolates tested for time period and number of studies are shown below the relevant time period.

**Figure 9:**
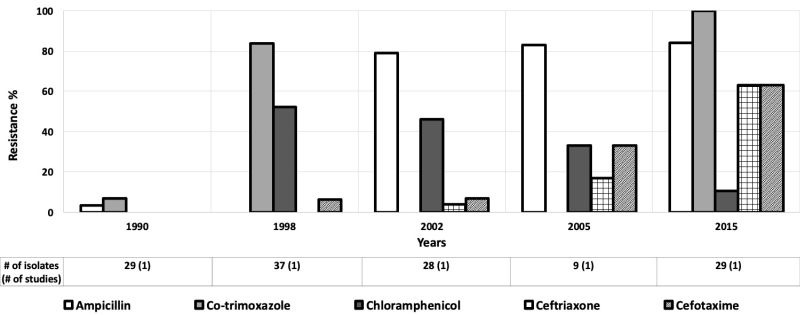
Antibiotic resistance among *Shigella* isolates in the Eastern Mediterranean Region (EMR) by time period. The number of isolates tested for time period and number of studies are shown below the relevant time period.

Nine studies assessed antibiotic resistance of *Shigella* isolates in children aged 0-5 years [31,32,34,44,49,51,53-55], and eight provided data for children aged 0-10 years [42,43,52,56-60] Resistance rates for ampicillin, co-trimoxazole, and chloramphenicol for *Shigella* in the AFR started at an alarmingly high rate ( ~ 90%) in 2006 and fluctuated over time (**Figure 8**) [34]. Ampicillin and co-trimoxazole resistance increased from ~ 7% in 1990 to around 90% to 100%, respectively by 2015 in EMR (**Figure 9**) [56,61]. Ceftriaxone resistance rose from 4% to 63% between 2002 and 2015 in the EMR [52,61]. Likewise, cefotaxime resistance increased from 7% to 63% from 2002 to 2015 [52,61]. Only these regions were included because of the data available for those regions were much greater than the other regions.

**Figure 10:**
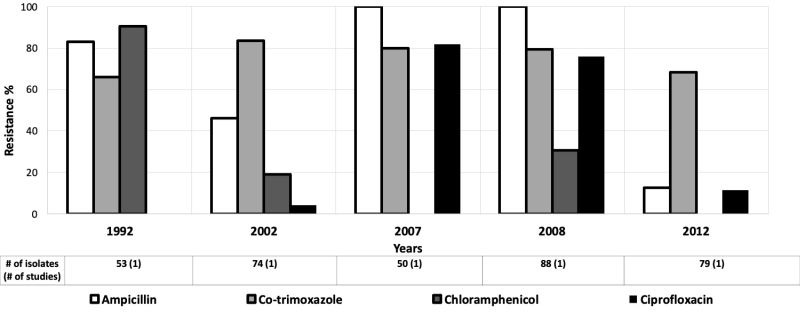
Antibiotic resistance among *Shigella* isolates in the South-East Asian Region (SEAR) by time period. The number of isolates tested for time period and number of studies are shown below the relevant time period.

In general, antibiotic resistant *Shigella* was highest in the SEAR. Resistance levels for ampicillin, co-trimoxazole and chloramphenicol hovered around 70% in SEAR in 1992 [60]. Subsequently, ampicillin resistance increased to as high as 100% [58,59], except two outlier studies in 2002 and 2012 (**Figure 10**) [43,58]. Co-trimoxazole resistance hovered around 70% from 1992 to 2012 [43,60]. Chloramphenicol resistance decreased to moderate levels ( ~ 30%) by 2008 [58]. Ciprofloxacin resistance started low at 4%, but rapidly increased to 76% by 2008 [58], except for one outlier in 2012 with low resistance at 11% (**Figure 10**) [43].

### Other enteric pathogens

#### Aeromonas

Two studies of antibiotic resistance of *Aeromonas* isolates included data for children aged 0-5 years [52,62]. According to Mansour and colleagues, in the EMR region, 52 isolates of *Aeromonas* were 99% resistant to ampicillin, 15% to nalidixic acid, 6% to chloramphenicol, 69% to cephalothin, and 39% to erythromycin during the period they studied [62]. In 2015, Tian et al. evaluated ten isolates of *Aeromonas* in the WPR region. They found that antibiotic resistance ranged from 10% to 40% (co-trimoxazole: 10%, cefotaxime: 30%, and tetracycline: 40%) [52]. Neither study evaluated the susceptibility of *Aeromonas* to fluoroquinolones.

#### Yersinia

Two studies of antibiotic-resistant *Yersinia* isolates provided data for children aged 0-10 years in EMR [63,64]. Both had small sample sizes. In 2002, Dallal and colleagues discovered 100% resistance of eight *Yersinia* isolates to ampicillin, penicillin, and cephalothin [63]. Another study of 16 *Yersinia* isolates found antibiotic resistance ranging from 50% to 94% (tetracycline: 50%, chloramphenicol: 63%, ciprofloxacin: 88%, and ceftriaxone: 94%) in 2006-2007 [64].

#### Vibrio cholerae

Two studies of *V. cholerae* provided data for children aged 0-5 years in the AFR. A 2011 study conducted in Dar es Salaam, Tanzania revealed antibiotic resistance levels ranging from 4.7% to 75% (cefuroxime: 4.7%; cephalothin: 31%, chloramphenicol: 69%, tetracycline: 72%, erythromycin: 75%, and ampicillin: 75%) for 16 isolates [34]. A 2017 recent study of 34 isolates from an outbreak in Lusaka, Zambia found antibiotic resistance ranging from 100% to co-trimoxazole; 32.4% to erythromycin, 26.5% to ciprofloxacin; 8.8% to chloramphenicol; and 0% for azithromycin, ampicillin, cefotaxime, and gentamicin [65].

#### Campylobacter

Seven studies provided data of antibiotic-resistant *Campylobacter* isolates [5,28,35,43,44,49,52]. Of these, one provided data for children aged 0-10 years [43]. These studies included 161 isolates from AFR, SEAR, and WPR. In India, Rathaur and colleagues analysed 17 isolates of *Campylobacter*. They found co-trimoxazole resistance was 53%, followed by ciprofloxacin (18%), then ampicillin (12%), and amoxicillin (5.8%). They did not assess macrolide resistance [43]. In Hawassa town, Ethiopia, Mulatu and colleagues found 20% resistance to co-trimoxazole, 10% to ciprofloxacin, and 55% to erythromycin [44].

Three studies in the WPR assessed resistant *Campylobacter* [28,49,52]. Thompson and colleagues discovered that ciprofloxacin resistance was 80%, whereas erythromycin resistance was 8% in Ho Chi Minh City, Vietnam, in 2009 [49]. In Wuhan, China, Tian and colleagues described resistance levels to ciprofloxacin and azithromycin as 60% and 6.7%, respectively in in 2015 [52]. In Shanghai in 2014, Chang and team found an even higher level (90%) of resistance to ciprofloxacin, whereas resistance to erythromycin and azithromycin was 11% [28].

## DISCUSSION

This systematic review yielded many valuable lessons about the global evolution of antibiotic resistance among common bacterial pathogens in LMICs during the last three decades. High-level resistance to older, inexpensive drugs like ampicillin, co-trimoxazole, and chloramphenicol is pervasive. Similarly, the last decade has witnessed alarming increases in resistance to broad-spectrum antibiotics, including third generation cephalosporins, especially ceftriaxone, and fluoroquinolones. Most studies evaluated ciprofloxacin and no other fluoroquinolones.

We did find variability between studies and regions. The increase in antibiotic resistance, especially for relatively newer antibiotics has implications for global [2] and national guidelines for the management of dysentery in vulnerable young age group. One example of such newer antibiotics includes ciprofloxacin (which is recommended by the WHO for treatment of invasive diarrhoea in children). Alternative oral options other than macrolides (azithromycin) or expensive parenteral agents such as aztreonam or carbapenems are limited. Thus, a need for continued surveillance of common bacterial enteropathogens among children of all ages and adults is urgent, as is the need for continued surveillance and effective communication of these results to clinicians with guidance about the optimal approach to managing invasive diarrhoea. We must also address widespread inappropriate antibiotic use for non-bloody diarrhoea in children [66,67] and adults [68].

As seen from the figures, there appear to be fluctuations in the resistance patterns to some antibiotics in a cyclic manner, with periods of rise and decline. This could be attributed potentially to several factors such as variations in widespread use of categories of antibiotics, inadequate infection control measures, and limited access to newer antibiotics.

The overuse and misuse of antibiotics can lead to the selection and spread of resistant bacteria, while poor infection control measures can facilitate their transmission. Additionally, the limited availability of newer antibiotics and the high cost of treatment may result in the increased use of older antibiotics, leading to the development and spread of resistance.

To combat antibiotic resistance, there needs to be a comprehensive approach that involves reducing unnecessary antibiotic use, improving infection control practices, and promoting the development and accessibility of new and effective antibiotics. Additionally, there should be increased efforts to monitor antibiotic resistance trends and implement targeted interventions to address local resistance patterns.

Although studies describing antibiotic resistance patterns in children with *V. cholerae* are limited, several outbreaks in adults have been found. A study in Nepal in 2011 looked at 836 isolates. Since 2006, 100% were resistant to nalidixic acid. Co-trimoxazole resistance remained constant in the 77%-100% range, whereas ciprofloxacin and tetracycline resistance peaked during 2010-2012 and then disappeared by 2016. Ampicillin resistance fluctuated over the last decade, increasing to 100% by 2016 [69].

Likewise, a study of cholera in Dhaka, Bangladesh in 2006 that included 13 isolates found resistance to tetracycline, erythromycin, ciprofloxacin, and azithromycin was increasing. Another similar study in Dhaka, Bangladesh was conducted in 2019, where 62 isolates were studied. About 89% of the isolates were resistant to ampicillin, 99% were resistant to erythromycin, almost all (99.7%) were resistant to trimethoprim-sulphamethoxazole, and 89% were resistant to ampicillin. In all cholera outbreaks, there should be susceptibility testing of a random selection of samples to assess resistance levels.

In 2015, a systematic review was conducted to investigate harmful practices in the management of childhood diarrhoea in LMICs. The review revealed that harmful practices, such as restricting fluids, breast milk, and food intake during diarrhoea, as well as incorrect use of antibiotics, were prevalent in countries with high diarrhoea-related mortality rates. The study identified health workers, relatives, community members, and traditional beliefs as the causes of these practices. This underscores the need to dispel the myths surrounding traditional diarrhoea management techniques among patients, health care workers, and communities [70].

Antibiotic misuse is a significant contributor to resistance, particularly in LMICs. Overuse of antibiotics for non-bloody diarrhoea is a well-known issue. In addition, widespread antibiotic misuse occurs for pulmonary diseases and malaria. A 2015 cross-sectional study in Zambia demonstrated that testing negative or not receiving a diagnosis of upper respiratory tract infection or malaria was linked to increased rates of antibiotic prescribing. This paper presents one example that demonstrates the overuse of antibiotics for common childhood infections including diarrhoea and respiratory tract infections while emphasizing the need to limit antibiotic use to patients with definite bacterial infections [68].

Overall, these studies emphasize the need to address harmful practices and inappropriate antibiotic use in LMICs. Efforts to improve the clinical management of febrile illnesses and strengthen interventions should be made to tackle this problem. This will require collaboration between health care workers, patients, and communities to dispel traditional beliefs and myths that perpetuate harmful practices [68,70].

### Limitations

This analysis has several limitations. First, there were few studies for several regions, including LMICs in Central America and North America (Mexico). Second, many studies did not evaluate susceptibility to important classes of antibiotics, including third generation cephalosporins, fluoroquinolones, and macrolides, or only studied some but not all of these antimicrobial agents. Third, many studies were limited to a specific city or region in a country and were therefore not generalizable to the whole country or WHO region. This presents challenges in comparing trends in resistance rates over time since the comparisons involve studies done in vastly different locations. This explains why there were some unusual rises and falls in the proportion of resistance to certain antibiotics. Fourth, there was a dearth of systematically collected antibiotic resistance data for less common bacterial pathogens. For instance, there were no relevant studies for *Plesiomonas.* Fifth, in case where several sources were used for calculating the mean values, we did not perform any initial statistical tests of comparing distribution characteristics, eg, ANOVA test. Therefore, some of the means we calculated may be void, as the corresponding sets of values derived from different works might be incomparable. Finally, only a limited number of studies included children aged six to 10 years.

Although this systematic review captured data from different regions, the information was collected over a wide range of time periods. The amount of information collected widely varied depending on the scope of the location where the data was collected. If the information was collected from a city, understandably, the number of isolates collected was smaller than if the study collected data from the entire country. Some studies tested the susceptibility of a few antibiotics, whereas other studies examined susceptibility for a higher number of antibiotics, making it challenging to draw fair comparisons between them. These limitations could be addressed by future surveillance programs, ideally using standardized microbiological methods, including testing the same panels of antibiotics and using global frameworks for development and stewardship to combat antimicrobial resistance as advised by the WHO [71].

## CONCLUSIONS

Resistance to inexpensive antibiotics for treating invasive diarrhoea in young children is widespread, and resistance to alternative treatments such as fluoroquinolones and later-generation cephalosporins are rising. We need better antimicrobial stewardship at multiple levels of health systems in LMICs to reduce the often-inappropriate use of antibiotics to treat community-acquired childhood diarrhoea and robust regional surveillance systems to track trends in antibiotic resistance.

Age stratified data on resistance of common causes of invasive diarrhoea (e.g. *Shigella* spp, *Salmonella* spp, and *Campylobacter* spp) to third generation cephalosporins, fluoroquinolones, and macrolides are needed from many regions of the world but especially in the AMR, EMR, and WPR. These need to include school-aged children and potentially adolescents in order to have a fuller picture of antibiotic resistance among diarrhoeal pathogens in older children. More data on resistance patterns among *Aeromonas*, *Yersinia*, *V. cholerae,* and *Campylobacter* are necessary to understand how the resistance patterns have been evolving in various regions. More studies should be done in AMR and EUR as most have been done in AFR and EMR.

## Additional material


Online Supplementary Document
