# Supplementary material for: Antibiotic resistance trends for common bacterial aetiologies of childhood diarrhoea in low- and middle-income countries: A systematic review
Source: J Glob Health. 2023 Jul 21;13:04060. doi: 10.7189/jogh.13.04060 (PMC10359834; doi:10.7189/jogh.13.04060)
Supplement: Online Supplementary Document [file jogh-13-04060-s001.pdf]

Bacteria	E coli						
**Region**	**Location (city, country)**	**Lead Author**	**Citation**	**Period of data collection**	**Mid-point year**	**Population studied (age of children in years)**	**Number of isolates**
	Maputo Province, Southern Mozambique	Mandomando I.M. et al	Mandomando, I. M., Macete, E. V., Ruiz, J., Sanz, S., Abacassamo, F., Vallès, X., Sacarlal, J., Navia, M. M., Vila, J., Alonso, P. L., & Gascon, J. (2007). Etiology of diarrhea in children younger than 5 years of age admitted in a rural hospital of southern Mozambique. *The American journal of tropical medicine and hygiene* , *76* (3), 522–527.	2000 - 2001	2000	0-5	94
	Dar es Salaam, Tanzania	Moyo S.J. et al	Moyo, S. J., Gro, N., Matee, M. I., Kitundu, J., Myrmel, H., Mylvaganam, H., Maselle, S. Y., & Langeland, N. (2011). Age specific aetiological agents of diarrhoea in hospitalized children aged less than five years in Dar es Salaam, Tanzania. *BMC pediatrics* , *11* , 19. https://doi.org/10.1186/1471-2431-11-19	2005 - 2006	2005	0-5	64
	Ghana	Djie-Maletz A. et al	Djie-Maletz, A., Reither, K., Danour, S., Anyidoho, L., Saad, E., Danikuu, F., Ziniel, P., Weitzel, T., Wagner, J., Bienzle, U., Stark, K., Seidu-Korkor, A., Mockenhaupt, F. P., & Ignatius, R. (2008). High rate of resistance to locally used antibiotics among enteric bacteria from children in Northern Ghana. *The Journal of antimicrobial chemotherapy* , *61* (6), 1315–1318. https://doi.org/10.1093/jac/dkn108	2005-2006	2005	0-4	318
AFR	Rural Tanzania: Kongwa District	Seidman J.C. et al	Seidman, J. C., Johnson, L. B., Levens, J., Mkocha, H., Muñoz, B., Silbergeld, E. K., West, S. K., & Coles, C. L. (2016). Longitudinal Comparison of Antibiotic Resistance in Diarrheagenic and Non-pathogenic Escherichia coli from Young Tanzanian Children. *Frontiers in microbiology* , *7* , 1420. https://doi.org/10.3389/fmicb.2016.01420	2009	2009	0-5	2492
	Maradi, Niger	Langendorf C. et al	Langendorf, C., Le Hello, S., Moumouni, A., Gouali, M., Mamaty, A. A., Grais, R. F., Weill, F. X., & Page, A. L. (2015). Enteric bacterial pathogens in children with diarrhea in Niger: diversity and antimicrobial resistance. *PloS one* , *10* (3), e0120275. https://doi.org/10.1371/journal.pone.0120275	2010 - 2012	2010	0-59 months	111
	Bahir Dar, Ethiopia	Adugna A. et al	Adugna, A., Kibret, M., Abera, B., Nibret, E., & Adal, M. (2015). Antibiogram of E. coli serotypes isolated from children aged under five with acute diarrhea in Bahir Dar town. *African health sciences* , *15* (2), 656–664. https://doi.org/10.4314/ahs.v15i2.45	2011 - 2012	2011	0-5	240
	Ouagadougou, Burkina Faso	Konate A. et al	Konaté, A., Dembélé, R., Guessennd, N. K., Kouadio, F. K., Kouadio, I. K., Ouattara, M. B., Kaboré, W., Kagambèga, A., Cissé, H., Ibrahim, H. B., Bagré, T. S., Traoré, A. S., & Barro, N. (2017). Epidemiology and Antibiotic Resistance Phenotypes of Diarrheagenic *Escherichia Coli* Responsible for Infantile Gastroenteritis in Ouagadougou, Burkina Faso. *European journal of microbiology & immunology* , *7* (3), 168–175. https://doi.org/10.1556/1886.2017.00014	2013-2015	2014	0-5	31
	Culican, Mexico	Uribe-Beltrán M.J. et al	Uribe-Beltrán, M. J., Ahumada-Santos, Y. P., Díaz-Camacho, S. P., Eslava-Campos, C. A., Reyes-Valenzuela, J. E., Báez-Flores, M. E., Osuna-Ramírez, I., & Delgado-Vargas, F. (2017). High prevalence of multidrug-resistant Escherichia coli isolates from children with and without diarrhoea and their susceptibility to the antibacterial activity of extracts/fractions of fruits native to Mexico. *Journal of medical microbiology* , *66* (7), 972–980. https://doi.org/10.1099/jmm.0.000548	2003-2004	2003	0-5	205
	Nicaragua	Amaya E. et al	Amaya, E., Reyes, D., Vilchez, S., Paniagua, M., Möllby, R., Nord, C. E., & Weintraub, A. (2011). Antibiotic resistance patterns of intestinal Escherichia coli isolates from Nicaraguan children. *Journal of medical microbiology* , *60* (Pt 2), 216–222. https://doi.org/10.1099/jmm.0.020842-0	2005-2006	2005	0-5	727
	Lima, Peru	Ochoa T.J. et al	Ochoa, T. J., Ruiz, J., Molina, M., Del Valle, L. J., Vargas, M., Gil, A. I., Ecker, L., Barletta, F., Hall, E., Cleary, T. G., & Lanata, C. F. (2009). High frequency of antimicrobial drug resistance of diarrheagenic Escherichia coli in infants in Peru. *The American journal of tropical medicine and hygiene* , *81* (2), 296–301.	2006 - 2007	2006	2-12 months	129
	Juiz de Fora, Brazil	Garcia P.G. et al	Garcia, P. G., Silva, V. L., & Diniz, C. G. (2011). Occurrence and antimicrobial drug susceptibility patterns of commensal and diarrheagenic Escherichia coli in fecal microbiota from children with and without acute diarrhea. *Journal of microbiology (Seoul, Korea)* , *49* (1), 46–52. https://doi.org/10.1007/s12275-011-0172-8	2007 - 2008	2007	0-5	136
AMR	La Paz, El Alto, and Cochabama, Bolivia	Gonzales L. et al	Gonzales, L., Joffre, E., Rivera, R., Sjöling, Å., Svennerholm, A. M., & Iñiguez, V. (2013). Prevalence, seasonality and severity of disease caused by pathogenic Escherichia coli in children with diarrhoea in Bolivia. *Journal of medical microbiology* , *62* (Pt 11), 1697–1706. https://doi.org/10.1099/jmm.0.060798-0	2007 - 2010	2008	<60 months	881
	Western Brazilian Amazon	Taborda, R.L.M. et al	Taborda, R.L., Silva, L.H., Orlandi, P.P., Batista, F.S., Rodrigues, R.S., & Matos, N.B. (2018). CHARACTERIZATION OF ENTEROAGGREGATIVE ESCHERICHIA COLI AMONG DIARRHEAL CHILDRENIN WESTERN BRAZILIAN AMAZON. *Arquivos de gastroenterologia, 55 4* , 390-396 .	2010 - 2012	2011	0-6	85
	Brazilian Amazon	Rodrigues R.S. et al	Rodrigues, R. S., Lima, N., Taborda, R., Esquerdo, R. P., Gama, A. R., Nogueira, P. A., Orlandi, P. P., & Matos, N. B. (2019). Antibiotic resistance and biofilm formation in children with Enteropathogenic Escherichia coli (EPEC) in Brazilian Amazon. *Journal of infection in developing countries* , *13* (8), 698–705. https://doi.org/10.3855/jidc.10674	2010 - 2012	2011	0-6	71
	Botucatu, Brazil	Dias R. et al	Dias, R. C., Dos Santos, B. C., Dos Santos, L. F., Vieira, M. A., Yamatogi, R. S., Mondelli, A. L., Sadatsune, T., Sforcin, J. M., Gomes, T. A., & Hernandes, R. T. (2016). Diarrheagenic Escherichia coli pathotypes investigation revealed atypical enteropathogenic E. coli as putative emerging diarrheal agents in children living in Botucatu, São Paulo State, Brazil. *APMIS : acta pathologica, microbiologica, et immunologica Scandinavica* , *124* (4), 299–308. https://doi.org/10.1111/apm.12501	2013-2014	2013	0-5	1,127

EMR	Azad Kashmir, Pakistan	Ahmed et al	Ahmed, Basharat & R, Shakoori & Ali, Syed & Shakoori, Abdul. (2009). Antimicrobial Resistance Pattern and Plasmid Analysis of Escherichia coli from Patients Suffering from Acute Diarrhoea in Azad Kashmir, Pakistan*. Pakistan journal of zoology. 41. 371-380.	1994 - 1998	1996	0-5	1210
	Hilla city, Iraq	Al-Saadi	Al-Saadi, Zainab & Tarish, Adnan & Saeed, Eiman. (2018). Phenotypic detection and antibiotics resistance pattern of local serotype of E. coli O157:H7 from children with acute diarrhea in Hilla city/ Iraq. Journal of Pharmaceutical Sciences and Research. 10. 604-609.	1995 - 1998	1996	0-5	55
	Rural Egypt: Abu Homos District, Beheira Governorate, Egypt, situated in the Nile Delta	Shaheen H. I. et al	Shaheen, H. I., Khalil, S. B., Rao, M. R., Abu Elyazeed, R., Wierzba, T. F., Peruski, L. F., Jr, Putnam, S., Navarro, A., Morsy, B. Z., Cravioto, A., Clemens, J. D., Svennerholm, A. M., & Savarino, S. J. (2004). Phenotypic profiles of enterotoxigenic Escherichia coli associated with early childhood diarrhea in rural Egypt. *Journal of clinical microbiology* , *42* (12), 5588–5595. https://doi.org/10.1128/JCM.42.12.5588-5595.2004	1995-1998	1996	0-3	859
	Yemen	Banajeh S. et al	Banajeh, S. M., Ba-Oum, N. H., & Al-Sanabani, R. M. (2001). Bacterial aetiology and anti-microbial resistance of childhood diarrhoea in Yemen. *Journal of tropical pediatrics* , *47* (5), 301–303. https://doi.org/10.1093/tropej/47.5.301	1998	1998	0-5	27
	Iran	Aslani M. et al	Aslani, M. M., Alikhani, M. Y., Zavari, A., Yousefi, R., & Zamani, A. R. (2011). Characterization of enteroaggregative Escherichia coli (EAEC) clinical isolates and their antibiotic resistance pattern. *International journal of infectious diseases : IJID : official publication of the International Society for Infectious Diseases* , *15* (2), e136–e139. https://doi.org/10.1016/j.ijid.2010.10.002	2007 - 2008	2007	0-12	15
	Kashan, Iran	Mitra M. et al	Motallebi, M., Piroozmand, A. & Rohani, M., Hosein, A. & Ahmad, K. (2011). Multiple drug resistance of enteropathogenic Escherichia coli isolated from children with diarrhea in Kashan, Iran. African Journal of Microbiology Research. 5. 10.5897/AJMR11.483.	2009 - 2010	2009	0-5	51
	Tehran, Iran	Heidary M. et al	Heidary, M., Momtaz, H., & Madani, M. (2014). Characterization of Diarrheagenic Antimicrobial Resistant Escherichia coli Isolated From Pediatric Patients in Tehran, Iran. *Iranian Red Crescent medical journal* , *16* (4), e12329. https://doi.org/10.5812/ircmj.12329	2010 - 2011	2010	0-5	154
	Tehran and Iilam in Iran	Karami P. et al	Karami, P., Bazmamoun, H., Sedighi, I., Mozaffari Nejad, A. S., Aslani, M. M., & Alikhani, M. Y. (2017). Antibacterial resistance patterns of extended spectrum β-lactamase - producing enteropathogenic Escherichia coli strains isolated from children. *Arab journal of gastroenterology : the official publication of the Pan-Arab Association of Gastroenterology* , *18* (4), 206–209. https://doi.org/10.1016/j.ajg.2017.11.004	2010 - 2012	2011	0-10	192
	Tabriz, Iran	Haghi F. et al	Haghi, F., Zeighami, H., Hajiahmadi, F., Khoshvaght, H., & Bayat, M. (2014). Frequency and antimicrobial resistance of diarrhoeagenic Escherichia coli from young children in Iran. *Journal of medical microbiology* , *63* (Pt 3), 427–432. https://doi.org/10.1099/jmm.0.064600-0	2011 - 2012	2011	0-5	140
	Terhan,Iran	Momtaz H. et al	Momtaz, H., Dehkordi, F.S., Hosseini, M.J. *et al.* Serogroups, virulence genes and antibiotic resistance in Shiga toxin-producing *Escherichia coli* isolated from diarrheic and non-diarrheic pediatric patients in Iran. *Gut Pathog* **5**, 39 (2013). https://doi.org/10.1186/1757-4749-5-39	2012 - 2013	2012	0-5	122
	Central Iran	Abbasi E. et al	Abbasi, E., Mondanizadeh, M., van Belkum, A., & Ghaznavi-Rad, E. (2020). Multi-Drug-Resistant Diarrheagenic *Escherichia coli* Pathotypes in Pediatric Patients with Gastroenteritis from Central Iran. *Infection and drug resistance* , *13* , 1387–1396. https://doi.org/10.2147/IDR.S247732	2015 - 2016	2015	0-10	91
	Wasit Province, Iraq	Abdul-hussein Z. et al	Abdul-hussein, Z., Raheema, R., & Insaf, A. (2018). Molecular Diagnosis of Diarrheagenic E. coli Infections Among the Pediatric Patients in Wasit Province, Iraq. Journal of Pure and Applied Microbiology. 12. 2229-2240. 10.22207/JPAM.12.4.62.	2017	2017	0-5	42
	Thi-qar, Iraq	Jabur S. G. et al	Sanaa Ghali Jabur1 , Murtada Hasan Abed2. (2020). Genetic Survey of Enteroaggregative E.coli in Diarrheic Children under 5 years in Thi-qar governorate. *Indian Journal of Forensic Medicine & Toxicology* , *14* (3), 1434–1439. https://doi.org/10.37506/ijfmt.v14i3.10609	2018 - 2019	2018	0-5	66
SEAR	Srinagar, India	Rathaur V. K. et al	Rathaur, V. K., Pathania, M., Jayara, A., & Yadav, N. (2014). Clinical study of acute childhood diarrhoea caused by bacterial enteropathogens. *Journal of clinical and diagnostic research : JCDR* , *8* (5), PC01–PC5. https://doi.org/10.7860/JCDR/2014/6677.4319	2012	2012	0-11	124
	Kanpur, India	Yadav M. et al	Yadav M, Bhatiani A, Kumar A, Bhaogliwal A, Sujatha R. Antimicrobial Resistance Pattern of Enteroaggregative Escherichia Coli isolated from acut diarrhea of pediatric patient under five in Kanpur region. Int J Pharm Sci Res. 2019;10:1902–5.	2017-2018	2017	0-5	40
WPR	Southeast China	Zheng S. et al	Zheng, S., Yu, F., Chen, X., Cui, D., Cheng, Y., Xie, G., Yang, X., Han, D., Wang, Y., Zhang, W., & Chen, Y. (2016). Enteropathogens in children less than 5 years of age with acute diarrhea: a 5-year surveillance study in the Southeast Coast of China. *BMC infectious diseases* , *16* (1), 434. https://doi.org/10.1186/s12879-016-1760-3	2009 - 2014	2011	0-5	177
	Shanghi, China	Chang H. et al	Chang, H., Zhang, L., Ge, Y., Cai, J., Wang, X., Huang, Z., Guo, J., Xu, H., Gu, Z., Chen, H., Xu, X., & Zeng, M. (2017). A Hospital-based Case-control Study of Diarrhea in Children in Shanghai. *The Pediatric infectious disease journal* , *36* (11), 1057–1063. https://doi.org/10.1097/INF.0000000000001562	2014	2014	0-5	201

Bacteria	Salmonella						
Region	Lead Author	Citation	Location (city, country)	Period of data collection	Midpoint Year	Population studied (age of children in years)	Number of isolates
AFR	Mandomando, I.M. et al	Mandomando, I. M., Macete, E. V., Ruiz, J., Sanz, S., Abacassamo, F., Vallès, X., Sacarlal, J., Navia, M. M., Vila, J., Alonso, P. L., & Gascon, J. (2007). Etiology of diarrhea in children younger than 5 years of age admitted in a rural hospital of southern Mozambique. *The American journal of tropical medicine and hygiene* , *76* (3), 522–527.	Maputo Province, Southern Mozambique	2000 - 2001	2000	0-5	13
AFR	Kariuki, S. et al	Kariuki, S., Revathi, G., Kariuki, N., Kiiru, J., Mwituria, J., & Hart, C. A. (2006). Characterisation of community acquired non-typhoidal Salmonella from bacteraemia and diarrhoeal infections in children admitted to hospital in Nairobi, Kenya. *BMC microbiology* , *6* , 101. https://doi.org/10.1186/1471-2180-6-101	Nairobi, Kenya	2002 - 2004	2003	4-84 months	162
AFR	Moyo, S.J. et al	Moyo, S.J., Gro, N., Matee, M.I. *et al.* Age specific aetiological agents of diarrhoea in hospitalized children aged less than five years in Dar es Salaam, Tanzania. *BMC Pediatr* **11,** 19 (2011). https://doi.org/10.1186/1471-2431-11-19	Dar es Salaam, Tanzania	2005 - 2006	2005	0-5	7
AFR	Beyene, G. er al	Beyene, G., Nair, S., Asrat, D., Mengistu, Y., Engers, H., & Wain, J. (2011). Multidrug resistant Salmonella Concord is a major cause of salmonellosis in children in Ethiopia. *Journal of infection in developing countries* , *5* (1), 23–33. https://doi.org/10.3855/jidc.906	Addis Ababa and Jimma, Ethiopia	2006	2006	6 months - 15 years	113
AFR	Bonkoungou, I.J.O. er al	Bonkoungou, I.J.O., Haukka, K., Österblad, M. *et al.* Bacterial and viral etiology of childhood diarrhea in Ouagadougou, Burkina Faso. *BMC Pediatr* **13,** 36 (2013). https://doi.org/10.1186/1471-2431-13-36	Ouagadougou, Burkina Faso	2009- 2010	2009	0-5	25
AFR	Langendorf, C. et al	Langendorf, C., Le Hello, S., Moumouni, A., Gouali, M., Mamaty, A. A., Grais, R. F., Weill, F. X., & Page, A. L. (2015). Enteric bacterial pathogens in children with diarrhea in Niger: diversity and antimicrobial resistance. *PloS one* , *10* (3), e0120275. https://doi.org/10.1371/journal.pone.0120275	Maradi, Niger	2010 - 2012	2011	0-5	360
AFR	Mulatu, G. et al	Mulatu, G., Beyene, G., & Zeynudin, A. (2014). Prevalence of Shigella, Salmonella and Campylobacter species and their susceptibility patters among under five children with diarrhea in Hawassa town, south Ethiopia. *Ethiopian journal of health sciences* , *24* (2), 101–108. https://doi.org/10.4314/ejhs.v24i2.1	Hawassa town, Ethiopia	2011	2011	0-5	4
AFR	Ameya, G. et al	Ameya, G., Tsalla, T., Getu, F., & Getu, E. (2018). Antimicrobial susceptibility pattern, and associated factors of Salmonella and Shigella infections among under five children in Arba Minch, South Ethiopia. *Annals of clinical microbiology and antimicrobials* , *17* (1), 1. https://doi.org/10.1186/s12941-018-0253-1	Arba Minch, Ethiopia	2017	2017	0-5	21
	Elamreen, A. et al	Abu Elamreen, F. H., Sharif, F. A., & Deeb, J. E. (2008). Isolation and antibiotic susceptibility of Salmonella and Shigella strains isolated from children in Gaza, Palestine from 1999 to 2006. Journal of gastroenterology and hepatology, 23(8 Pt 2), e330–e333. https://doi.org/10.1111/j.1440-1746.2007.05139.x	Gaza, Palestine	1999 - 2006	2002	1 month - 12 years	65

EMR	Banajeh, S. et al	Banajeh, S. M., Ba-Oum, N. H., & Al-Sanabani, R. M. (2001). Bacterial aetiology and anti-microbial resistance of childhood diarrhoea in Yemen. *Journal of tropical pediatrics* , *47* (5), 301–303. https://doi.org/10.1093/tropej/47.5.301	Sanaa, Yemen	1998	1998	0-5	38
	Elamreen, A et al	Abu Elamreen, F. H., Abed, A. A., & Sharif, F. A. (2007). Detection and identification of bacterial enteropathogens by polymerase chain reaction and conventional techniques in childhood acute gastroenteritis in Gaza, Palestine. *International journal of infectious diseases : IJID : official publication of the International Society for Infectious Diseases* , *11* (6), 501–507. https://doi.org/10.1016/j.ijid.2007.01.010	Gaza, Palestine	2005	2005	0-5	3
	Harb, A et al	Harb, A., O'Dea, M., Hanan, Z. K., Abraham, S., & Habib, I. (2017). Prevalence, risk factors and antimicrobial resistance of Salmonella diarrhoeal infection among children in Thi-Qar Governorate, Iraq. *Epidemiology and infection* , *145* (16), 3486–3496. https://doi.org/10.1017/S0950268817002400	Thi-Qar Governorate, Iraq	2016	2016	0-5	33
SEAR	Rathaur, V.K. et al	Rathaur, V. K., Pathania, M., Jayara, A., & Yadav, N. (2014). Clinical study of acute childhood diarrhoea caused by bacterial enteropathogens. *Journal of clinical and diagnostic research : JCDR* , *8* (5), PC01–PC5. https://doi.org/10.7860/JCDR/2014/6677.4319	Srinagar, India	2012	2012	0-11	38
WPR	Thompson C.N., et al	Thompson CN, Phan MV, Hoang NV, et al. A prospective multi-center observational study of children hospitalized with diarrhea in Ho Chi Minh City, Vietnam. Am J Trop Med Hyg. 2015;92(5):1045-1052. doi:10.4269/ajtmh.14-0655	Ho Chi Minh, Vietnam	2002-2004	2003	0-5	57
	Qu M., et al	Qu M, Lv B, Zhang X, et al. Prevalence and antibiotic resistance of bacterial pathogens isolated from childhood diarrhea in Beijing, China (2010-2014). Gut Pathog. 2016;8:31. Published 2016 Jun 13. doi:10.1186/s13099-016-0116-2	Beijing, China	2010-2014	2012	0-5	109
	Tian, L. et al	Tian L, Zhu X, Chen Z, et al. Characteristics of bacterial pathogens associated with acute diarrhea in children under 5 years of age: a hospital-based cross-sectional study. BMC Infect Dis. 2016;16:253. Published 2016 Jun 7. doi:10.1186/s12879-016-1603-2	Wuhan, China	2014 - 2015	2014	0-5	43
	Chang H., et al	Chang H, Zhang L, Ge Y, et al. A Hospital-based Case-control Study of Diarrhea in Children in Shanghai. Pediatr Infect Dis J. 2017;36(11):1057-1063. doi:10.1097/INF.0000000000001562	Shanghi, China	2014	2014	0-5	73
	Liang, B. et al	Liang B, Xie Y, He S, et al. Prevalence, serotypes, and drug resistance of nontyphoidal Salmonella among paediatric patients in a tertiary hospital in Guangzhou, China, 2014-2016. J Infect Public Health. 2019;12(2):252-257. doi:10.1016/j.jiph.2018.10.012	Guangzhou, China	2014-2016	2015	6 month - 3 years	220

Bacteria	Shigella						
**Region**	**Location (City, Country)**	**Lead Author**	**Citation**	**Period of data collection**	**Midpoint year**	**Population studied (age of children in years)**	**Number of isolates**
AFR	Dar es Salaam, Tanzania	Moyo, S.J. et al	Moyo, S. J., Gro, N., Matee, M. I., Kitundu, J., Myrmel, H., Mylvaganam, H., Maselle, S. Y., & Langeland, N. (2011). Age specific aetiological agents of diarrhoea in hospitalized children aged less than five years in Dar es Salaam, Tanzania. *BMC pediatrics* , *11* , 19. https://doi.org/10.1186/1471-2431-11-19	2005 - 2006	2005	0-5	15
	Ouagadougou, Burkina Faso	Bonkoungo u, I.J. et al	Bonkoungou, I. J., Haukka, K., Österblad, M., Hakanen, A. J., Traoré, A. S., Barro, N., & Siitonen, A. (2013). Bacterial and viral etiology of childhood diarrhea in Ouagadougou, Burkina Faso. *BMC pediatrics* , *13* , 36. https://doi.org/10.1186/1471-2431-13-36	2009 - 2010	2009	0-5	16
	Hawassa Town, Ethiopia	Mulatu, G. et al	Mulatu, G., Beyene, G., & Zeynudin, A. (2014). Prevalence of Shigella, Salmonella and Campylobacter species and their susceptibility patters among under five children with diarrhea in Hawassa town, south Ethiopia. *Ethiopian journal of health sciences* , *24* (2), 101–108. https://doi.org/10.4314/ejhs.v24i2.1	2011	2011	0-5	11
	Maradi, Niger	Langendorf C. et al	Langendorf, C., Le Hello, S., Moumouni, A., Gouali, M., Mamaty, A. A., Grais, R. F., Weill, F. X., & Page, A. L. (2015). Enteric bacterial pathogens in children with diarrhea in Niger: diversity and antimicrobial resistance. *PloS one* , *10* (3), e0120275. https://doi.org/10.1371/journal.pone.0120275	2011	2011	0-5	138
	Arba Minch,Ethiopia	Ameya, G. et al	Ameya, G., Tsalla, T., Getu, F., & Getu, E. (2018). Antimicrobial susceptibility pattern, and associated factors of Salmonella and Shigella infections among under five children in Arba Minch, South Ethiopia. *Annals of clinical microbiology and antimicrobials* , *17* (1), 1. https://doi.org/10.1186/s12941-018-0253-1	2017	2017	0-5	8
AMR	Lima, Peru	Lluque, A. et al	Lluque, A., Mosquito, S., Gomes, C., Riveros, M., Durand, D., Tilley, D. H., Bernal, M., Prada, A., Ochoa, T. J., & Ruiz, J. (2015). Virulence factors and mechanisms of antimicrobial resistance in Shigella strains from periurban areas of Lima (Peru). *International journal of medical microbiology : IJMM* , *305* (4-5), 480–490. https://doi.org/10.1016/j.ijmm.2015.04.005	2010 - 2012	2011	0-2	56
EMR	Lahore, Pakistan	Khalil, K. et al	Khalil, K., Khan, S. R., Mazhar, K., Kaijser, B., & Lindblom, G. B. (1998). Occurrence and susceptibility to antibiotics of Shigella species in stools of hospitalized children with bloody diarrhea in Pakistan. *The American journal of tropical medicine and hygiene* , *58* (6), 800–803. https://doi.org/10.4269/ajtmh.1998.58.800	1990	1990	1 - 72 months	29
	Sanaa, Yemen	Banajeh, S. M. et al	Banajeh, S. M., Ba-Oum, N. H., & Al-Sanabani, R. M. (2001). Bacterial aetiology and anti-microbial resistance of childhood diarrhoea in Yemen. *Journal of tropical pediatrics* , *47* (5), 301–303. https://doi.org/10.1093/tropej/47.5.301	1998	1998	0-5	37
	Gaza, Palestine	Elamreen, A. et al	Abu Elamreen, F. H., Sharif, F. A., & Deeb, J. E. (2008). Isolation and antibiotic susceptibility of Salmonella and Shigella strains isolated from children in Gaza, Palestine from 1999 to 2006. *Journal of gastroenterology and hepatology* , *23* (8 Pt 2), e330–e333. https://doi.org/10.1111/j.1440-1746.2007.05139.x	2002	2002	1 month - 12 years	28
	Gaza, Palestine	Elamreen, A. et al	Abu Elamreen, F. H., Abed, A. A., & Sharif, F. A. (2007). Detection and identification of bacterial enteropathogens by polymerase chain reaction and conventional techniques in childhood acute gastroenteritis in Gaza, Palestine. *International journal of infectious diseases : IJID : official publication of the International Society for Infectious Diseases* , *11* (6), 501–507. https://doi.org/10.1016/j.ijid.2007.01.010	2005	2005	0-5	9

	Central Iran	Abbasi, E. et al	Abbasi, E., Abtahi, H., van Belkum, A., & Ghaznavi-Rad, E. (2019). Multidrug-resistant *Shigella* infection in pediatric patients with diarrhea from central Iran. *Infection and drug resistance* , *12* , 1535–1544. https://doi.org/10.2147/IDR.S203654	2015	2015	1-16	19
EUR	Ankara, Turkey	Aysev, A. D. et al	Aysev, A. D., & Guriz, H. (1998). Drug resistance of Shigella strains isolated in Ankara, Turkey, 1993-1996. *Scandinavian journal of infectious diseases* , *30* (4), 351–353. https://doi.org/10.1080/00365549850160620	2011	2011	9 months - 15 years	289
SEAR	Andaman Islands, India	Bhattacharya D. et al	Bhattacharya, D., Bhattacharya, H., Sayi, D. S., Bharadwaj, A. P., Singhania, M., Sugunan, A. P., & Roy, S. (2015). Changing patterns and widening of antibiotic resistance in Shigella spp. over a decade (2000-2011), Andaman Islands, India. *Epidemiology and infection* , *143* (3), 470–477. https://doi.org/10.1017/S0950268814000958	2000 - 2005	2002	0-14	74
	Andaman Islands, India	Bhattacharya D. et al	Bhattacharya, D., Bhattacharya, H., Sayi, D. S., Bharadwaj, A. P., Singhania, M., Sugunan, A. P., & Roy, S. (2015). Changing patterns and widening of antibiotic resistance in Shigella spp. over a decade (2000-2011), Andaman Islands, India. *Epidemiology and infection* , *143* (3), 470–477. https://doi.org/10.1017/S0950268814000958	2006-2011	2008	0-14	88
	Srinagar, India	Rathaur, V. K. et al	Rathaur, V. K., Pathania, M., Jayara, A., & Yadav, N. (2014). Clinical study of acute childhood diarrhoea caused by bacterial enteropathogens. *Journal of clinical and diagnostic research : JCDR* , *8* (5), PC01–PC5. https://doi.org/10.7860/JCDR/2014/6677.4319	2005	2005	0-11	79
	Chandigarh, India	Thapa B.R. et al	Thapa, B. R., Ventkateswarlu, K., Malik, A. K., & Panigrahi, D. (1995). Shigellosis in children from north India: a clinicopathological study. *Journal of tropical pediatrics* , *41* (5), 303–307. https://doi.org/10.1093/tropej/41.5.303	2005 - 2006	2005	0-12	53
	Andaman Islands, India	Bhattacharya, D. et al	Bhattacharya, D., Sugunan, A. P., Bhattacharjee, H., Thamizhmani, R., Sayi, D. S., Thanasekaran, K., Manimunda, S. P., Ghosh, A. R., Bharadwaj, A. P., Singhania, M., & Roy, S. (2012). Antimicrobial resistance in Shigella--rapid increase & widening of spectrum in Andaman Islands, India. *The Indian journal of medical research* , *135* (3), 365–370.	2006 - 2009	2007	6 months - 14 years	50
WPR	Ho Chi Minh City, Vietnam	Thompson C.N. et al	Thompson, C. N., Phan, M. V., Hoang, N. V., Minh, P. V., Vinh, N. T., Thuy, C. T., Nga, T. T., Rabaa, M. A., Duy, P. T., Dung, T. T., Phat, V. V., Nga, T. V., Tu, l., Tuyen, H. T., Yoshihara, K., Jenkins, C., Duong, V. T., Phuc, H. L., Tuyet, P. T., Ngoc, N. M., ... Baker, S. (2015). A prospective multi-center observational study of children hospitalized with diarrhea in Ho Chi Minh City, Vietnam. *The American journal of tropical medicine and hygiene* , *92* (5), 1045–1052. https://doi.org/10.4269/ajtmh.14-0655	2009-2010	2009	0-5	62

Bacteria	Campylobacter						
**Region**	**Authors**	**Citation**	**Location (city, country)**	**Years of data collection**	**Midpoint Year**	**Population studied (age of children in years)**	**Number of isolates**
AFR	Aboderin A. O., et al	Aboderin, A. O., Smith, S. I., Oyelese, A. O., Onipede, A. O., Zailani, S. B., & Coker, A. O. (2002). Role of Campylobacter jejuni/coli in diarrhoea in Ile-Ife, Nigeria. *East African medical journal* , *79* (8), 423–426.	Ile-Ife, Nigeria	1981 - 1983	1982	0-5	31
	Mandomando, I.M. et al	Mandomando, I. M., Macete, E. V., Ruiz, J., Sanz, S., Abacassamo, F., Vallès, X., Sacarlal, J., Navia, M. M., Vila, J., Alonso, P. L., & Gascon, J. (2007). Etiology of diarrhea in children younger than 5 years of age admitted in a rural hospital of southern Mozambique. *The American journal of tropical medicine and hygiene* , *76* (3), 522–527.	Maputo Province, Southern Mozambique	2000 - 2001	2000	0-5	9
	Mulatu, G. et al	Mulatu, G., Beyene, G., & Zeynudin, A. (2014). Prevalence of Shigella, Salmonella and Campylobacter species and their susceptibility patters among under five children with diarrhea in Hawassa town, south Ethiopia. *Ethiopian journal of health sciences* , *24* (2), 101–108. https://doi.org/10.4314/ejhs.v24i2.1	Hawassa town, Ethiopia	2011	2011	0-5	20
SEAR	Rathaur, V. K. et al	Rathaur, V. K., Pathania, M., Jayara, A., & Yadav, N. (2014). Clinical study of acute childhood diarrhoea caused by bacterial enteropathogens. *Journal of clinical and diagnostic research : JCDR* , *8* (5), PC01–PC5. https://doi.org/10.7860/JCDR/2014/6677.4319	Srinagar, India	2005	2005	0-11	17
WPR	Thompson C.N., et al	Thompson, C. N., Phan, M. V., Hoang, N. V., Minh, P. V., Vinh, N. T., Thuy, C. T., Nga, T. T., Rabaa, M. A., Duy, P. T., Dung, T. T., Phat, V. V., Nga, T. V., Tu, l., Tuyen, H. T., Yoshihara, K., Jenkins, C., Duong, V. T., Phuc, H. L., Tuyet, P. T., Ngoc, N. M., … Baker, S. (2015). A prospective multi-center observational study of children hospitalized with diarrhea in Ho Chi Minh City, Vietnam. *The American journal of tropical medicine and hygiene* , *92* (5), 1045–1052. https://doi.org/10.4269/ajtmh.14-0655	Ho Chi Minh, Vietnam	2009 - 2010	2009	0-5	31
	Tian L., et al	Tian, L., Zhu, X., Chen, Z., Liu, W., Li, S., Yu, W., Zhang, W., Xiang, X., & Sun, Z. (2016). Characteristics of bacterial pathogens associated with acute diarrhea in children under 5 years of age: a hospital-based cross-sectional study. *BMC infectious diseases* , *16* , 253. https://doi.org/10.1186/s12879-016-1603-2	Wuhan, China	2014 - 2015	2014	0-5	15
	Chang H., et al	Chang, H., Zhang, L., Ge, Y., Cai, J., Wang, X., Huang, Z., Guo, J., Xu, H., Gu, Z., Chen, H., Xu, X., & Zeng, M. (2017). A Hospital-based Case-control Study of Diarrhea in Children in Shanghai. *The Pediatric infectious disease journal* , *36* (11), 1057–1063. https://doi.org/10.1097/INF.0000000000001562	Shanghi, China	2014	2014	0-5	38

Bacteria	Aeromonas						
**Region**	**Authors**	**Citation**	**Location (city, country)**	**Years of data collection**	**Midpoint Year**	**Population studied (age of children in years)**	**Number of isolates**
EMR	Mansour, A. M. et al	Mansour, A. M., Abd Elkhalek, R., Shaheen, H. I., El Mohammady, H., Refaey, S., Hassan, K., Riddle, M., Sanders, J. W., Sebeny, P. J., Young, S. Y., & Frenck, R. (2012). Burden of Aeromonas hydrophila-associated diarrhea among children younger than 2 years in rural Egyptian community. *Journal of infection in developing countries* , *6* (12), 842–846. https://doi.org/10.3855/jidc.2390	Rural Egypt	2004 - 2007	2005	0-2	52
WPR	Tian, L. et al	Tian, L., Zhu, X., Chen, Z., Liu, W., Li, S., Yu, W., Zhang, W., Xiang, X., & Sun, Z. (2016). Characteristics of bacterial pathogens associated with acute diarrhea in children under 5 years of age: a hospital-based cross-sectional study. *BMC infectious diseases* , *16* , 253. https://doi.org/10.1186/s12879-016-1603-2	Wuhan, China	2014 - 2015	2014	0-5	10

Bacteria	Yersinia						
**Region**	**Authors**	**Citation**	**Location (city, country)**	**Years of data collection**	**Midpoint Year**	**Population studied (age of children in years)**	**Number of isolates**
EMR	Soltan-Dallal, M. M. et al	Soltan-Dallal, M. M., & Moezardalan, K. (2004). Frequency of Yersinia species infection in paediatric acute diarrhoea in Tehran. *Eastern Mediterranean health journal = La revue de sante de la Mediterranee orientale = al-Majallah al-sihhiyah li-sharq al-mutawassit* , *10* (1-2), 152–158.	Tehran, Iran	2002	2002	0-12	8
	El Qouqa, I. A. et al	El Qouqa, I. A., El Jarou, M. A., Samaha, A. S., Al Afifi, A. S., & Al Jarousha, A. M. (2011). Yersinia enterocolitica infection among children aged less than 12 years: a case-control study. *International journal of infectious diseases : IJID : official publication of the International Society for Infectious Diseases* , *15* (1), e48–e53. https://doi.org/10.1016/j.ijid.2010.09.010	Gaza, Palestine	2006 - 2007	2006	0-12	16

Bacteria	Vibrio Cholera						
**Region**	**Location (city, country)**	**Lead author**	**Citation**	**Years of data collection**	**Midpoint Year**	**Population studied (age of children in years)**	**Number of isolates**
AFR	Dar es Salaam, Tanzania	Moyo, S.J. et al	Moyo, S. J., Gro, N., Matee, M. I., Kitundu, J., Myrmel, H., Mylvaganam, H., Maselle, S. Y., & Langeland, N. (2011). Age specific aetiological agents of diarrhoea in hospitalized children aged less than five years in Dar es Salaam, Tanzania. *BMC pediatrics* , *11* , 19. https://doi.org/10.1186/1471-2431-11-19	2005-2006	2005	0-5	16
